# Effects of overnight fasting on handgrip strength in inpatients

**DOI:** 10.31744/einstein_journal/2019AO4418

**Published:** 2018-12-28

**Authors:** Wesley Santana Correa-Arruda, Iara dos Anjos Vaez, José Eduardo Aguilar-Nascimento, Diana Borges Dock-Nascimento

**Affiliations:** 1 Programa de Pós-Graduação, Faculdade de Ciências Médicas, Universidade Federal de Mato Grosso, Cuiabá, MT, Brazil

**Keywords:** Muscle strength, Fasting, Diet, Nutritional status, Malnutrition, Força muscular, Jejum, Dieta, Estado nutricional, Desnutrição

## Abstract

**Objective:**

To investigate the effects of overnight fasting on handgrip strength of adult inpatients.

**Methods:**

A prospective clinical study enrolling 221 adult patients. The endpoints were handgrip strength obtained by dynamometry in three time points (morning after an overnight fasting, after breakfast and after lunch) and the cumulative handgrip strength (mean of handgrip strength after breakfast and lunch) in the same day. The mean of three handgrip strength measures was considered to represent each time point. A cut-off for the mean overnight fasting handgrip strength at the 50^th^ percentile (35.5kg for males and 27.7kg for females) was used for comparisons. We registered the age, sex, current and usual weight (kg), weight loss (kg), diagnosis of cancer, nutritional status, elderly frequency, digestive tract symptoms, type of oral diet, and the amount of dinner ingested the night before handgrip strength (zero intake, until 50%, <100% and 100%).

**Results:**

Handgrip strength evaluated after an overnight fasting (31.2±8.7kg) was lesser when compared with handgrip strength after breakfast (31.6±8.8kg; p=0.01), and with cumulative handgrip strength (31.7±8.8kg; p<0.001). Handgrip strength was greater in patients who ingested 100% (33.2±9.1kg *versus* 30.4±8.4kg; p=0.03) and above 50% of dinner (32.1±8.4kg *versus* 28.6±8.8kg; p=0.006). Multivariate analysis showed that ingesting below 50% of dinner, severe malnutrition, and elderly were independent factors for handgrip strength reduction after overnight fasting.

**Conclusion:**

The muscular function was impaired after an overnight fasting of adult patients hospitalized for medical treatment, especially for those with low ingestion, malnourished and elderly.

## RESUMO

**Objetivo:**

Investigar os efeitos do jejum noturno na força muscular de pacientes adultos internados.

**Métodos:**

Estudo clínico prospectivo realizado com 221 pacientes adultos. Os desfechos observados foram a força de preensão palmar avaliada pela dinamometria em jejum, após o desjejum, após o almoço, e acumulada (média da força de preensão palmar após desjejum e almoço). Foi considerada a média das três medidas da força de preensão palmar para representar cada momento. O ponto de corte para a média da força de preensão palmar em jejum categorizada no percentil 50 (35,5kg para homens, e 27,7kg para mulheres) foi usado para as comparações. Investigaram-se idade, sexo, peso atual e habitual (kg), perda de peso (kg), diagnóstico de câncer, estado nutricional, frequência de idosos, sintomas do trato digestório, tipo de dieta oral e quantidade ingerida no jantar no dia anterior à mensuração da força de preensão palmar (ingestão zero, até 50%, <100 e 100%).

**Resultados:**

A força de preensão palmar após o jejum noturno foi menor que a força após o desjejum (31,1±8,7kg *versus* 31,6±8,8kg; p=0,01) e quando foi considerada a força acumulada (31,7±8,8kg; p<0,001). A força de preensão palmar em jejum foi maior para os pacientes que ingeriram 100% (33,2±9,1kg *versus* 30,4±8,4kg; p=0,03) e >50% do jantar (32,1±8,4kg *versus* 28,6±8,8kg; p=0,006). A análise multivariada mostrou que ingerir menos da metade do jantar, desnutrição grave e ser idoso foram fatores independentes para redução da força de preensão palmar em jejum.

**Conclusão:**

A função muscular ficou comprometida após jejum noturno em pacientes adultos internados para tratamento clínico, especialmente nos casos de baixa ingestão alimentar, desnutridos e idosos.

## INTRODUCTION

For the last few decades, several studies have shown alarming rates of hospital malnutrition in Brazil and worldwide. Disease-related malnutrition has not decreased over the years, and about 20 to 50% of patients are malnourished upon admission or become malnourished during their hospital stay.^(^ ^1^ ^–^ ^3^ ^)^ Despite advanced techniques of patient-care, malnutrition is still underdiagnosed and undertreated in hospitals, which leads to increased complications, readmissions and mortality.^(^ ^4^ ^,^ ^5^ ^)^ In this context, several factors can further complicate malnutrition, such as a low consumption of the available meals and pre-procedure fasting.

Hospital diets are important because they guarantee nutritional support and preserve and/or reinstate the patient's nutritional status. However, patients do not always eat all the food they are given. Studies have shown that approximately 30 to 50% of food patients receive is discarded.^(^ ^6^ ^,^ ^7^ ^)^ A Polish study showed that the 30-day risk mortality was 6.1 times higher for patients with a reduced nutrition intake during the previous week, and patients who reported a zero intake faced a risk that was 7.6 times higher.^(^ ^7^ ^)^

Fasting before surgeries, exams and procedures is another factor that contributes to hospital malnutrition.^(^ ^8^ ^,^ ^9^ ^)^ Studies have questioned this traditional recommendation of prolonged fasting,^(^ ^10^ ^,^ ^11^ ^)^ which increases metabolic stress, insulin-resistance, discomfort, nutritional and other complications, and death.^(^ ^12^ ^,^ ^13^ ^)^ Franklin et al., showed that, of the 22.6% of patients on prolonged fasting or liquid diets for 3 days, fasting was adequate in only 58.6% of cases.^(^ ^14^ ^)^ Another study found that patients were fasting for 14 hours before endoscopies and 58 hours after surgeries. These practices show that the fasting period is always longer than prescribed and recommended.^(^ ^15^ ^)^

Sorita et al., found that 46.6% of patients were prescribed at least one period of fasting, with a mean duration of 12.8 hours.^(^ ^16^ ^)^ That resulted in the absence of two (17.5%) meals per day. The frequent prescription for fasting is based on a precaution, a “just in case” for possible procedures.

Therefore, to reduce and treat hospital malnutrition, it is paramount to identify complications in the patient's nutritional status and detect the condition early.^(^ ^17^ ^,^ ^18^ ^)^ Among the assessment methods of a patient's nutritional status, the handgrip strength test is simple, non-invasive, low-cost and detects strength loss or gain in a short period of time.^(^ ^19^ ^)^

Dynamometry obtained by handgrip strength (HGS) is a good indicator of complications, length of hospital stay, and mortality.^(^ ^20^ ^)^ With this technique we can assess, within days or hours, functional and nutritional alterations and the efficacy of the nutritional therapy prescribed.^(^ ^21^ ^)^

## OBJECTIVE

To investigate the effects of overnight fasting on the muscle strength of adult patients admitted for medical treatments.

## METHODS

This is a prospective clinical study that included 221 patients admitted to the medical clinic of *Hospital Universitário Júlio* Müller (HUJM) in the Brazilian city of Cuiabá (State of Mato Grosso) between May 2015 and June 2017. The study was approved by the Research Ethics Committee of the institution, under protocol number 920.942, CAAE: 34901014.7.0000.5541. We included patients under clinical treatment. All participants signed an informed consent form. Data were collected by the researcher up to 48 hours after admission.

We excluded any patients with edema, painful symptoms, or any disease that prevented the measurement of HGS or dominant upper limb strength, and those who had had food or water before the evaluation.

The main variable was HGS (kg), measured after overnight fasting (fasting HGS), after breakfast (breakfast HGS) and after lunch (lunch HGS). We calculated the mean HGS obtained after breakfast and lunch (accumulated HGS). Mean fasting HGS was categorized in the 50^th^ percentile at 35.5kg for male and 27.7kg for female patients.

Handgrip strength was determined by a hydraulic dynamometer (Saehan Corporation, Masan, Korea^®^). Patients were in the seating position with feet planted on the ground, dominant arm parallel to the torso, elbow flexed at 90°, forearm and wrist in neutral positions, and were requested to exert maximum force at once. Three measurements were taken at 1-minute intervals. All three measurements were recorded, and the mean value was calculated.

The following data were also evaluated: age, sex, current weight (kg), usual weight, weight loss (kg), diagnosis of cancer, nutritional status by subjective global assessment (SGA), frequency of elderly individuals (≥60 years), digestive tract symptoms (intestinal constipation, diarrhea, nausea, abdominal distention and intestinal bleeding in the last 3 days), type of oral diet prescribed (semi-liquid/soft and bland/normal) and approximated amount of dinner intake the night before the HGS test. The amounts were categorized as zero intake (no consumption at all), 50% intake (categorized for the statistical analysis as >0 to ≤50%), intake over 50%, but not all (categorized for the statistical analysis as >50 and <100%) and 100% intake. This variable was collected directly with the patient through an interview in the morning of the test day. We compared the effect of the amount of dinner intake to the results of the breakfast HGS (first measurement).

Well-nourished patients were classified as SGA-A. Those at risk of malnutrition or moderately malnourished were classified as SGA-B. Patients with severe malnourishment were classified as SGA-C.^(^ ^22^ ^)^ Patients who were severely or moderately malnourished, or at risk of malnutrition were considered malnourished.

### Statistical analysis

For categorical variables, we used the χ^2^ test or Fisher's exact test. Homogenous variables and those with normal distribution were analyzed by Student's *t* test. The paired *t* test was used to compare fasting HGS, breakfast and lunch HGS and accumulated HGS. The multivariate analysis was done through the binary logistic regression using significant variables (p<0.20) and through the χ^2^ test with HGS categorized in 50 percentile for males (35.33kg) and females (27.7kg). To avoid collinearity, the variables ‘weight loss’ and ‘malnourished’ were removed for the multivariate analysis. Results were expressed in mean and standard deviation (SD). Significance was established at 5% (p<0.05). We used the Statistical Package for Social Sciences (SPSS), version 20.0.

## RESULTS

A total of 160 patients were excluded because they did not sign the informed consent form or ingested water or food before the test. We had a total of 221 participants with a mean age of 56±16 years. There were 93 (42.1%) elderly patients and 128 (57.9%) non-elderly adults, and 119 (53.8%) of participants were male. Of the participants, 28 (12.7%) were admitted for oncological treatment and 193 (87.3%) for clinical treatment. The participants’ mean current body weight was 65.4±14.9kg and their mean usual body weight was 71.1±15.1kg. As to nutrition status, 38 (17.2%) patients were eutrophic (SGA-A), 69 (31.2%) presented risk of malnutrition or were moderately malnourished (SGA-B), and 114 (51.6%) were severely malnourished (SGA-C).

**Figure 1:**
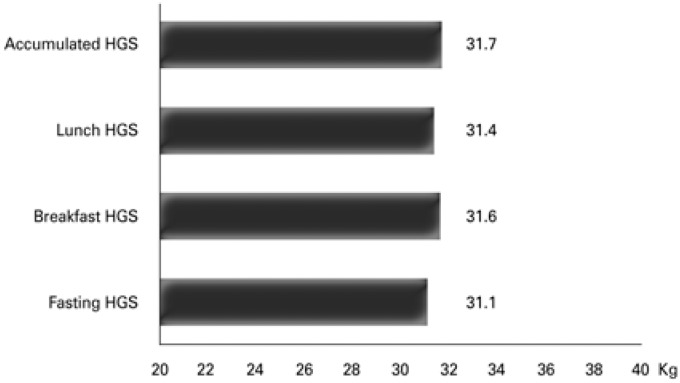
Handgrip strength during the three moments of testing and the accumulated force among the studied patients. Values expressed as means HGS: handgrip strength.

**Figure 2:**
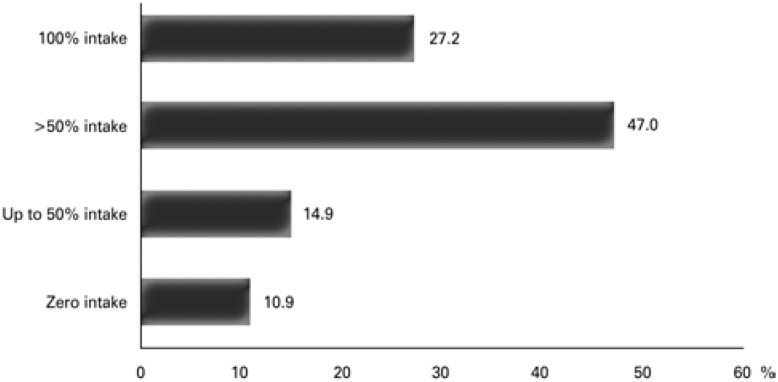
Food intake at dinner the night before handgrip strength measurements

Fasting HGS was 31.1±8.7kg. Breakfast HGS was 31.6±8.8kg. Lunch HGS was 31.4±8.5kg. Accumulated HGS was 31.7±8.8kg (Figure 1). There was zero intake at dinner for 24 (10.9%) patients; 33 patients (14.9%) had an intake of up to 50%; 104 (47.0%) had >50%; and 60 (27.2%) had a 100% intake (Figure 2).

**Figure 3:**
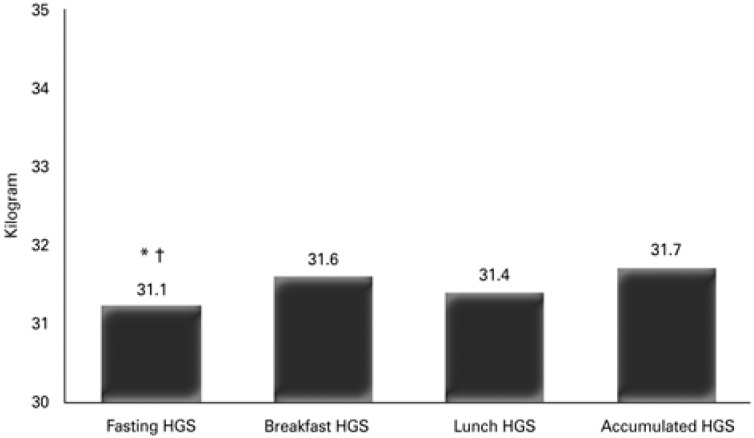
Comparison of fasting, breakfast, lunch and accumulated handgrip strength among the studied patients * p=0.01 *versus* breakfast HGS; ^†^ p<0.001 *versus* accumulated HGS. HGS: handgrip strength.

**Figure 4:**
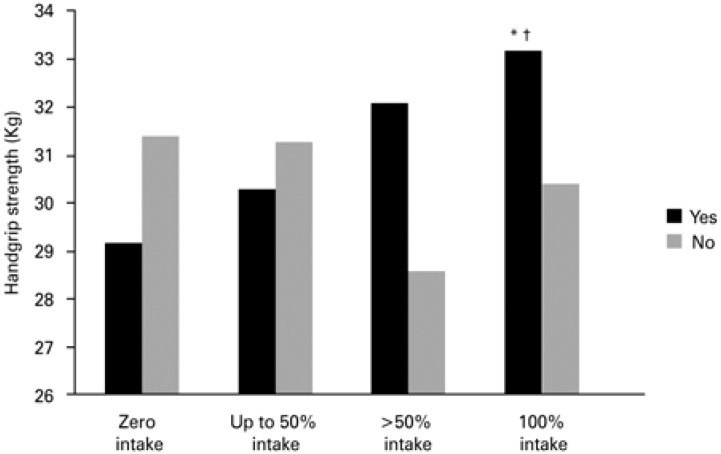
Comparison between fasting handgrip strength according to the amount of dinner intake the night before the measurement * p=0.006 *versus* intake <50%; ^†^ p=0.03 *versus* intake <100%.

Fasting HGS was lower than breakfast HGS (31.1±8.7kg *versus* 31.6±8.8kg; p=0.01). There was a difference between fasting HGS and accumulated HGS (31.1±8.7kg *versus* 31.7±8.8kg; p<0.001). No difference was found between fasting HGS and lunch HGS (p=0.16) (Figure 3). There was an increase in fasting HGS for patients with 100% dinner intake (33.2±9.1kg *versus* 30.4±8.4kg; p=0.03) and >50% dinner intake (32.1±8.4kg *versus* 28.6±8.8kg; p=0.006). No alteration was found in fasting HGS for patients with up to 50% intake (p=0.52) and zero intake (p=0.24) (Figure 4).

Patients with an intake of up to 50% (p=0.007); severely malnourished patients (p=0.009); malnourished patients (p=0.01); those with weight-loss (p=0.03); and elderly patients (p=0.03) presented an increased risk of reduced HGS after fasting (Table 1). The multivariate analysis showed that a <50% dinner intake, severe malnourishment, and age ≥60 years were independent factors for a lower fasting HGS (Table 2).

**Table 1:** Presence of fasting average grip strength at the 50th percentile (male and female) according to the variables studied

Variables	Frequency	*Odds ratio*	95%CI	p value
Up to 50% dinner intake	39/109 *versus* 22/112	2.28	1.24-4.19	0.007
Severely malnourished	66/109 *versus* 48/112	1.41	1.08-1.83	0.009
Malnourished	76/137 *versus* 33/84	1.28	1.03-1.58	0.019
Weight loss	81/109 *versus* 68/112	1.22	1.01-1.47	0.031
Elderly (≥60 years)	55/109 *versus* 38/112	1.49	1.08-2.04	0.031
Type of oral diet	38/109 *versus* 30/112	1.30	0.87-1.94	0.193
GIT symptoms	27/109 *versus* 20/112	1.38	0.79-2.90	0.209
Females	50/109 *versus* 52/112	1.01	0.79-1.29	0.934
Oncologic	12/109 *versus* 16/112	1.34	0.60-2.99	0.464

95%CI: 95% confidence interval; GIT: gastrointestinal.

**Table 2:** Independent risk factors determined by multivariate analysis for lower fasting handgrip strength

Risk factors	*Odds ratio*	95%CI	p value
Up to 50% dinner intake	2.17	1.16-4.06	0.018
Severe malnutrition	1.86	1.06-3.26	0.028
Elderly (age ≥60 years)	1.98	1.12-3.50	0.019
Type of oral diet	1.15	0.62-2.13	0.655
Digestive symptom	1.12	0.55-2.29	0.739

95%CI: 95% confidence interval.

## DISCUSSION

Results have shown that overnight fasting reduced HGS, which increased after food intake. A study has shown that HGS after pre-operative overnight fasting was lower in comparison to the HGS of patients who had a liquid intake 2 hours before surgery.^(^ ^21^ ^)^ The literature shows that fasting is harmful and that a dietary intake, even if only liquid, can improve HGS.^(^ ^23^ ^,^ ^24^ ^)^

Our study showed that, on its own, overnight fasting reduces patients’ strength. It is well known that energy expenditure at night is lower then during the day. During a night's sleep, we are fasting and in basal metabolism – during the day, however, our metabolism is working at full capacity. That means that fasting during the day is more harmful, because it increases the expenditure of reserves.^(^ ^25^ ^)^

Therefore, the routinely prescribed fasting before surgeries, exams, and procedures increases metabolic stress. This type of fasting is more harmful because the patient is awake, under stress and hungry. We can then state, with base on physiological facts, that fasting during the day is more damaging to muscular strength than overnight fasting. We must consider that, if the patient's strength is reduced by overnight fasting, it is even more reduced by routine fasting prescriptions in hospitals.

On the other hand, the inadequate selection of food in hospitals is also frequent. Additionally, fasting is often prescribed erroneously, and patients spend long periods without receiving any nutrients, in situations in which they could be receiving a low residue diet.^(^ ^25^ ^)^

In this study, we found that the amount of dinner intake negatively affected HGS – patients who had had a larger dinner intake presented a better HGS. A study with 3,122 patients showed that 23% of them had an intake <25%. These patients were older, had more severe conditions and were hospitalized for longer periods than those whose intake was >50%.^(^ ^26^ ^)^

A low-calorie intake is frequent among hospitalized patients. Approximately 30% of meals are rejected and discarded,^(^ ^27^ ^)^ which increases the rates of malnutrition and complications. According to Kondrup,^(^ ^28^ ^)^ hospital malnutrition can be attributed to inefficient hospital meal services. That includes a dissatisfactory quality of the food and a lack of flexibility regarding menu improvements. The poor quality of the food and lack of staff training are some of the causes for this reduced food intake.^(^ ^29^ ^)^

A low food intake during one week before admission is an independent factor for nutritional risk.^(^ ^6^ ^,^ ^30^ ^)^ On the other hand, about 25 to 50% of patients are malnourished upon admission,^(^ ^4^ ^)^ and this reduced intake hinders their nutritional status even further.

In our study, more than 50% of patients were severely malnourished, and malnutrition almost doubled the chances of a reduced HGS. Our findings showed that a dinner intake of <50% is an independent factor for the reduction of HGS.

Our study is a warning against the damages to the functional capacity determined by the muscle strength suffered by patients who are prescribed long periods of fasting and have a low intake of the hospital diet. Therefore, proactive measures are required to optimize fasting times and increase hospital food intake. These measures, together with continuous training for the multiprofessional team, can reduce hospital malnutrition, complications, re-admissions, and mortality.

## CONCLUSION

Muscle strength determined by handgrip strength was found to be compromised after overnight fasting in adult patients admitted for clinical treatments, especially in patients with a low food intake, those who were malnourished and elderly patients.
